# Cyril George Russ Wood

**Published:** 1938

**Authors:** 


### Obituary

### CYRIL GEORGE RUSS WOOD, O.B.E., F.R.C.S.

It is with great regret that we chronicle the death of Cyril George Russ Wood on 26th September at the age of 69. He was the only son of Mr. Cyril J. Wood, of Bath. He entered the Medical School of University College, Bristol, in 1885, qualified M.R.C.S., L.R.C.P. in 1892 and passed the F.R.C.S. (Eng.) in 1902. He was an Infirmary student and married the daughter of one of the B.R.I. surgeons, Charles Steele. Stimulated by the advice and example of Mr. Richardson Cross, he decided to specialize in ophthalmology, and after holding ophthalmic appointments in Southport he became Honorary Surgeon to the Eye, Ear and Throat Hospital, Shrewsbury, and Honorary Ophthalmic and Aural Surgeon to the Royal Salop Infirmary, as well as several other hospitals in the vicinity of Shrewsbury.

Russ Wood was one of the founders of the Oxford Ophthalmological Congress, and held the office of honorary secretary in 1928, and of Master in 1935. He was President of the Midland Ophthalmological Society. When he retired from practice in Shrewsbury in 1931 he was appointed Assistant Surgeon and Pathologist to the Oxford Eye Hospital and later Consulting Surgeon. He was a lecturer in the Oxford postgraduate course in ophthalmology and examiner in ophthalmology at Queen's University, Belfast.

Mr. Cyril Walker writes of him as follows :—

"Though I knew Russ Wood forty years ago, when he was working at the Bristol Eye Hospital, it was not until his appointment as secretary to the Oxford Ophthalmological Congress that I had an opportunity of appreciating his sterling qualities. When, in 1928, Bernard Cridland resigned the secretaryship and became "Master" of the Congress, I remember it came as somewhat of a surprise to me that he said he knew just the man to succeed him—Russ Wood. Events fully justified this prediction. He always had the interests of the Congress at heart, and with his alert and active mind he rarely lost an opportunity of securing a contribution from anyone who had been doing important ophthalmic work.

"One of the things which struck me most about Russ Wood was his invariable consideration for the wishes and feelings of others. During my term of office as Master of the Congress he frequently invited my opinion on various questions, but I always felt that he had fully considered the matter and that his judgment was to be relied upon. It was always a pleasure to me to recognize his rather sprawly handwriting, for I knew at once that the letter was from a sincere and valued friend."

